# AlphaScreen-based homogeneous assay using a pair of 25-residue artificial proteins for high-throughput analysis of non-native IgG

**DOI:** 10.1038/s41598-017-12693-w

**Published:** 2017-09-29

**Authors:** Yukako Senga, Hiroshi Imamura, Takamitsu Miyafusa, Hideki Watanabe, Shinya Honda

**Affiliations:** Biomedical Research Institute, National Institute of Advanced Industrial Science and Technology (AIST), 1-1-1 Higashi, Tsukuba, Ibaraki, 305-8566 Japan

## Abstract

Therapeutic IgG becomes unstable under various stresses in the manufacturing process. The resulting non-native IgG molecules tend to associate with each other and form aggregates. Because such aggregates not only decrease the pharmacological effect but also become a potential risk factor for immunogenicity, rapid analysis of aggregation is required for quality control of therapeutic IgG. In this study, we developed a homogeneous assay using AlphaScreen and AF.2A1. AF.2A1 is a 25-residue artificial protein that binds specifically to non-native IgG generated under chemical and physical stresses. This assay is performed in a short period of time. Our results show that AF.2A1-AlphaScreen may be used to evaluate the various types of IgG, as AF.2A1 recognizes the non-native structure in the constant region (Fc region) of IgG. The assay was effective for detection of non-native IgG, with particle size up to ca. 500 nm, generated under acid, heat, and stirring conditions. In addition, this technique is suitable for analyzing non-native IgG in CHO cell culture supernatant and mixed with large amounts of native IgG. These results indicate the potential of AF.2A1-AlphaScreen to be used as a high-throughput evaluation method for process monitoring as well as quality testing in the manufacturing of therapeutic IgG.

## Introduction

Therapeutic immunoglobulin G (IgG) has been widely used to treat cancer and inflammatory diseases, and its market is large and growing rapidly. Therapeutic IgG is a pharmaceutical product made of protein, which is less stable than small molecular weight chemical drugs. Proteins unfold with changes in temperature, pH, or pressure, and unfolded proteins tend to associate with each other and form aggregates. Indeed, aggregates of therapeutic IgG are formed as by-products in the manufacturing process^1,2^. When therapeutic IgG is exposed to stress factors in the manufacturing process such as acidic pH, freeze/thaw, and mechanical stress (shaking or stirring), it forms various types of non-native IgG in terms of shape and size^3–5^. If non-native monomers are present in therapeutic IgG productions, aggregates may form during the storage period. In this study, the word “non-native” indicates the state in which a molecule loses native tertiary structure regardless of the particle size of aggregates^6,7^. “Non-native IgG” includes monomers, oligomers, small aggregates, and large aggregates^4,5,8,9^. Being “aggregate” is defined here as a state in which multiple molecules of native or non-native proteins are associated. In general, native IgG partially or fully unfolds under stress conditions. Combined with other molecules, non-native molecules form oligomers. In many cases, small aggregates are generated, based on which large aggregates are produced^10–12^. Such aggregates not only decrease the pharmacological effect but also become a major factor for immunogenicity^13–15^. Hence, it is important to evaluate the quality of therapeutic IgG by analyzing non-native IgG, especially non-native monomers, oligomers, and small aggregates.

In a previous study, we developed AF.2A1, a 25-residue artificial protein, which is useful for detecting non-native IgG with a structural change in the constant region (Fc region)^16^. AF.2A1 binds efficiently to the Fc region of non-native IgG generated under chemical or physical stresses but not to native IgG^16,17^. Based on the structural changes caused by these stresses, AF.2A1 discriminates between non-native and native IgG^17^. AF.2A1 exhibits nanomolar affinity to the Fc region of IgG and shows an exquisite conformational specificity. In the previous study, Watanabe *et al*. performed a surface plasmon resonance assay to detect non-native IgG, and it was suggested that AF.2A1 has the potential for versatile application to a wide variety of assays^17^. To develop an automated assay system using AF.2A1 to analyze non-native IgG in the manufacturing of therapeutic IgG, it is necessary to include multi-analyte evaluation and high-throughput screening.

To characterize non-native IgG, a variety of methods have been used, including size exclusion chromatography (SEC), dynamic light scattering (DLS), field flow fractionation, analytical ultracentrifugation, nanoparticle tracking analysis, resonance mass measurement, flow imaging microscopy, nuclear magnetic resonance, circular dichroism, fluorescence, infrared, and turbidity^18,19^. Although all these methods give useful information based on their measurement principles, each has some methodological limitations^17^. Hence, a measuring method should be developed that can achieve the following five points: (1) high-throughput measurements, (2) a short period of time needed to obtain results, (3) easy evaluation of trace amounts of contamination, (4) measurement from cell culture supernatants, and (5) high specificity for the target.

To meet the above requisites, we focused on the AlphaScreen technique, which is an alternative approach to enzyme-linked immunosorbent assay (ELISA), because it enables easy-to-use and high-throughput analysis^20,21^. Historically, ELISA has been a typical method to detect and quantify biomolecules in heterogeneous samples^22^. However, it takes a long time to perform ELISA because of its washing steps, and it is difficult to adapt it to high-throughput operation and automation. In contrast, the advantage of AlphaScreen lies in an “addition only” no-wash format^18^. Because AlphaScreen is a no-wash and homogeneous bead-based sandwich immunoassay, it eliminates the washing and blocking steps required for plate-based immunoassays such as ELISA that often result in analyte dilution and human errors^23^.

In this study, we aimed to develop an assay method for high-throughput detection of non-native IgG in a short time period by combining AF.2A1 with the AlphaScreen technique. The usefulness of AF.2A1-AlphaScreen was investigated using different types of IgG under different stress conditions. Furthermore, we attempted to detect non-native IgG in crude extracts, cell culture supernatants, and mixed with large amounts of native IgG. This paper discusses the advantages of the AF.2A1-AlphaScreen technique for process monitoring and quality testing of therapeutic IgG.

## Results

### Examining the detectability of non-native IgG in large amounts of native IgG

**Figure 1:**
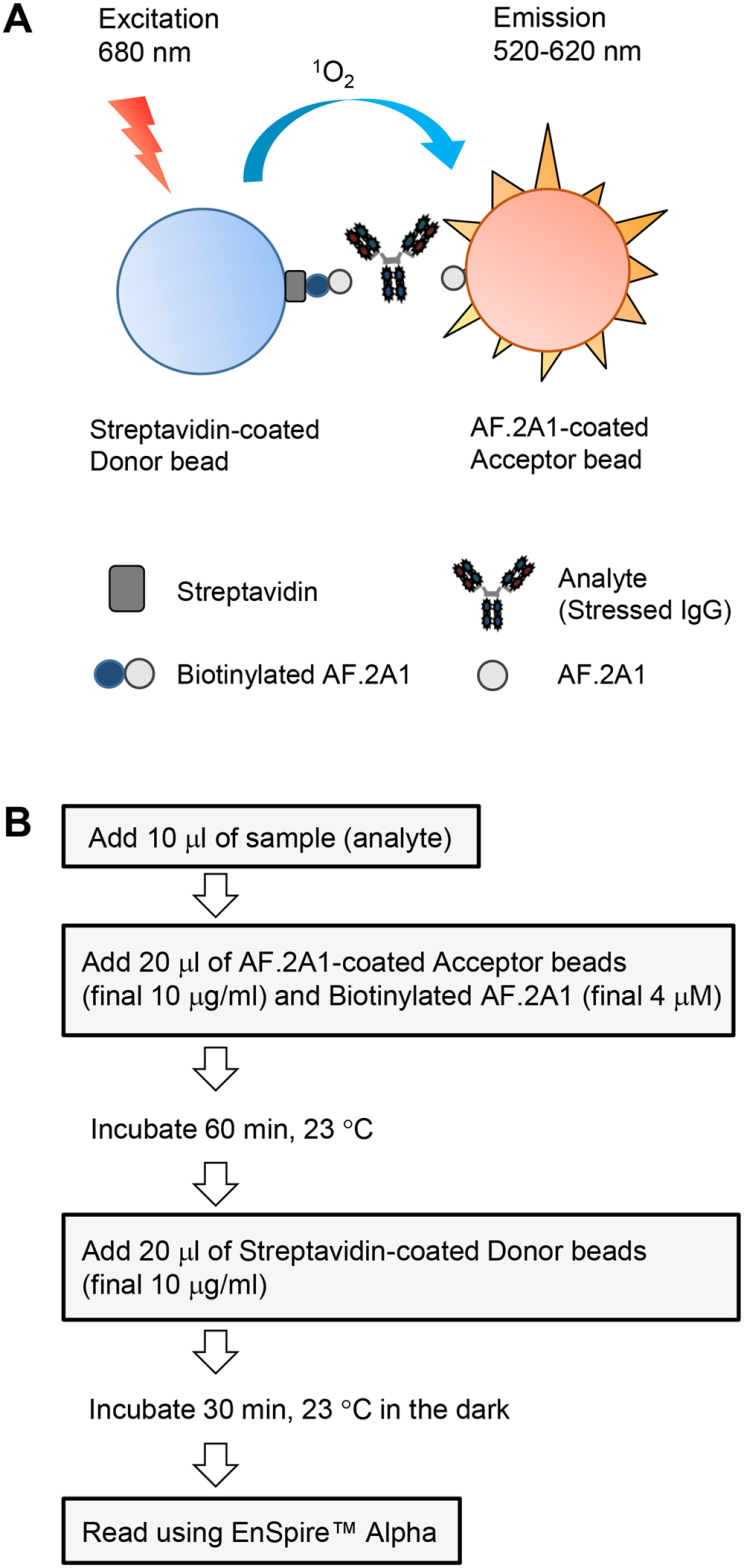
Principle of the AF.2A1-AlphaScreen technique. (**A**) Schematic illustration of AlphaScreen for detection of non-native IgG. The detection of non-native IgG is enabled by the sandwich structure involving a pair of AF.2A1 proteins. One is chemically conjugated to acceptor beads and the other is biotinylated, which is captured by streptavidin donor beads. The AlphaScreen signal is generated when donor beads are brought into close proximity with acceptor beads, allowing transfer of a singlet oxygen from the photo-excited donor to the acceptor. (**B**) Procedure of the AF.2A1-AlphaScreen technique.

**Figure 2:**
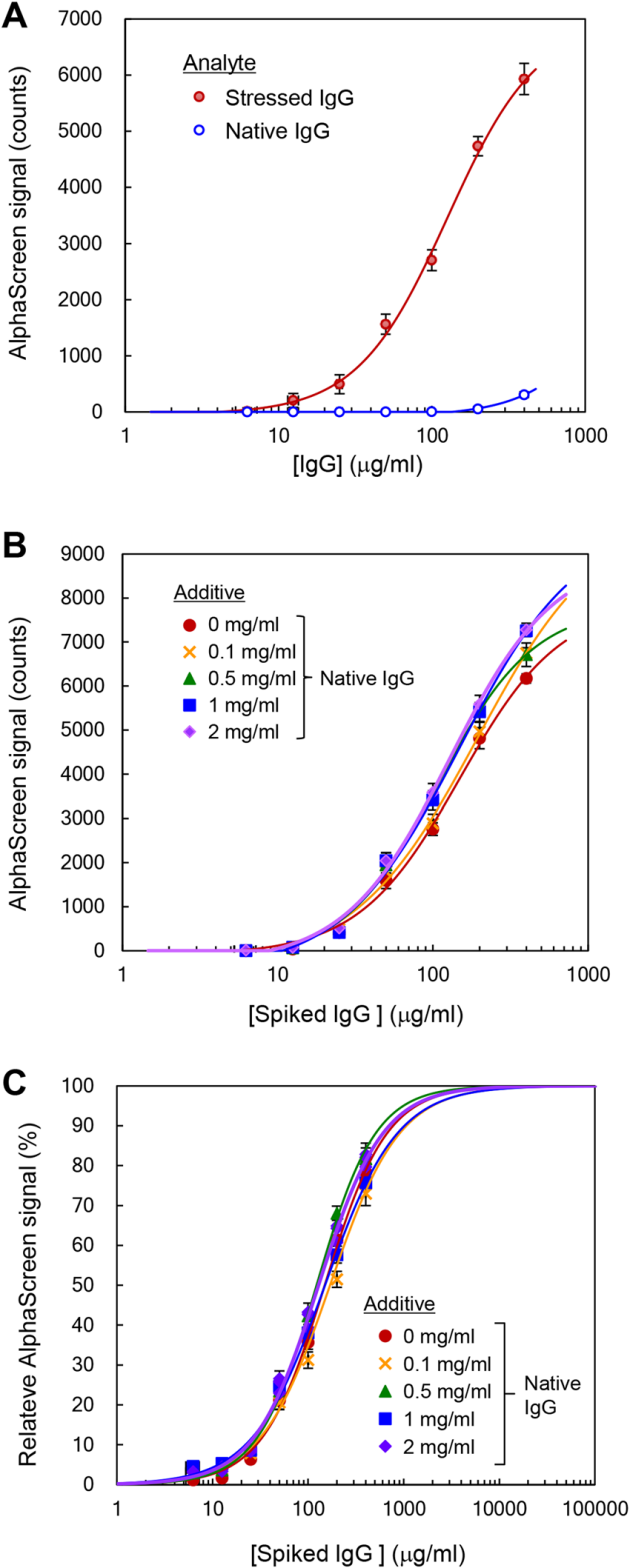
Detection of non-native IgG using the AF.2A1-AlphaScreen technique. (**A**) Comparison of stressed IgG and native IgG responses. Stressed-IgG was diluted with PBS. (**B**) Detection of non-native IgG spiked into a solution of native IgG. Stressed IgG was prepared as in (**A**). Stressed-IgG was diluted with PBS: 0.1 mg/ml, 0.5 mg/ml, 1 mg/ml, and 2 mg/ml mAb_A (native IgG). (**C**) Relative intensity calculated using the parameters computed in (**B**). Three independent experiments were performed, and the data were presented as mean ± standard deviation (SD). MAb_A was used in this experiment.

To determine the sensitivity of AF.2A1-AlphaScreen and the dynamic range of quantification for non-native IgG, we generated a standard curve using acid-stressed IgG serially diluted in phosphate-buffered saline (PBS). The AlphaScreen signal was generated when streptavidin-coated donor beads were brought in close proximity to AF.2A1-coated acceptor beads through binding between biotin-AF.2A1 on the donor bead, non-native IgG, and AF.2A1 on the acceptor bead. (Fig. 1A and B). Before measurements, we prepared a sample of acid-stressed IgG by dialyzing monoclonal IgG (mAb_A was used; see Table 1) against 100 mM glycine-HCl buffer solution (pH 2.0) at 4 °C, followed by neutralization. Note that native IgG is antibody without stress-treatment. As can be seen in Fig. 2A, the AlphaScreen signal gradually increased in parallel with the amount of acid-stressed IgG. The limits of detection (LOD) and limits of quantitation (LOQ) were 8.5 μg/ml and 25.6 μg/ml, respectively (see Methods).

**Table 1:** Characteristics of IgG1 monoclonal antibodies (mAb_A, mAb_B, and mAb_C).

	Isotype	Species	Molecular weight (kDa)	Isoelectric point
mAb_A	IgG1 kappa	mouse-human chimeric	145	8.7
mAb_B	IgG1 kappa	humanized	148	8.5
mAb_C	IgG1 kappa	humanized	149	8.1

If the AF.2A1-AlphaScreen is used for quality testing of therapeutic IgG, a large amount of native IgG co-existing in solution may become an obstacle. We therefore determined whether non-native IgG was detected using AF.2A1-AlphaScreen even in the presence of large amounts of native IgG. We prepared measurement samples by spiking acid-stressed mAb_A into PBS or into highly concentrated solutions of native IgG. Comparison of results from different conditions revealed that non-native IgG was successfully detected when native IgG was abundant in solution (Fig. 2B and C). Figure 2C shows the relative intensity normalized by setting the maximum AlphaScreen signal as 100% of the reactivity between AF.2A1 and non-native IgG. The concentration corresponding to 50% of the effective dose (ED50) was 142 ± 7 μg/ml when acid-stressed IgG was spiked into the PBS buffer. The ED50 values of acid-stressed IgG, which was spiked into solutions containing native IgG at 0.1 mg/ml, 0.5 mg/ml, 1 mg/ml, and 2 mg/ml, were 168 ± 7 μg/ml, 124 ± 6 μg/ml, 150 ± 4 μg/ml, and 128 ± 2 μg/ml, respectively. The ED50 of samples after spiking acid-stressed IgG into highly concentrated solutions of native IgG were within ± 20% of that of samples in PBS. These data suggest that errors in the determined values fall within that range, even if a calibration curve prepared only with stressed IgG (red circle in Fig. 2B) is used for quantification. The interaction between AF.2A1 and non-native IgG is similar in the presence or absence of large amounts of native IgG (Fig. 2C). Furthermore, it is sufficient to dilute the sample to at least 2 mg/ml. The LOD corresponding to approximately 0.1% of native IgG concentration indicates that AF.2A1-AlphaScreen has a higher sensitivity than conventional ones such as SEC and DLS.

Note that stress such as acid treatment not only increases the concentration of non-native IgG molecules, but also yields refolded, native IgG molecules^4,5^. Thus, it should be borne in mind that the concentrations of IgG (designated in the figures as [IgG], [spiked IgG], [stressed IgG]) do not represent the concentration of non-native IgG, but the total concentration of IgG molecules ( = [non-native IgG] + [native IgG]). As will be discussed later, the concentration balance between non-native and native molecules depends on the stability of the IgG subjected to stress.

### Examining the applicability of AF.2A1-AlphaScreen to various types of IgG in detecting non-native structures

**Figure 3:**
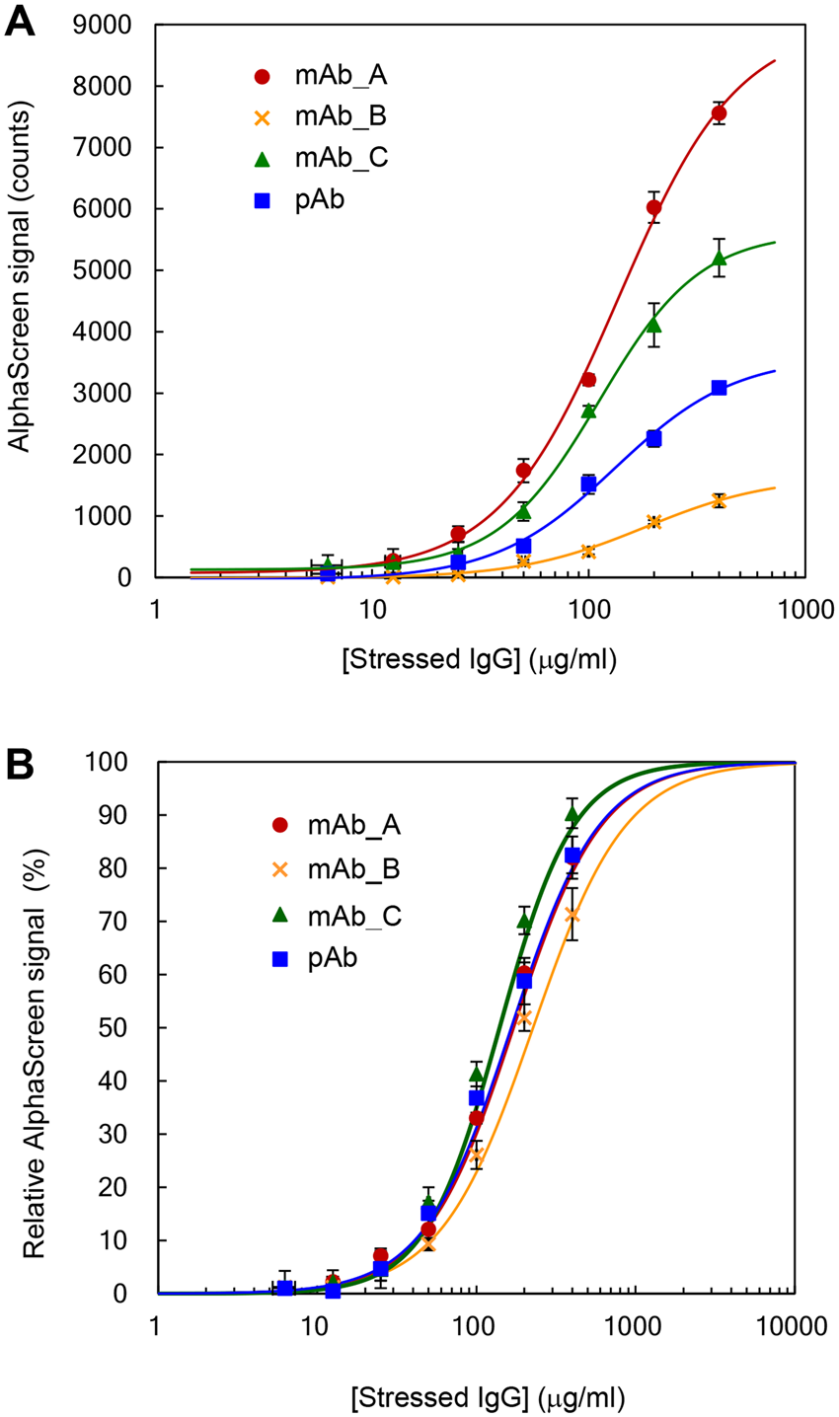
Comparing stress responses of different types of IgG. (**A**) Comparison between mAb_A, mAb_B, mAb_C, and pAb. Each IgG was diluted with PBS. (**B**) Relative intensity calculated using the parameters computed in (**A**). Three independent experiments were performed, and the data were presented as mean ± SD.

Because various types of IgG have been developed as therapeutic agents, we determined whether our technique was able to detect different types of IgG. To compare the AF.2A1-AlphaScreen signals, we used three types of monoclonal IgG1 and a polyclonal IgG (mAb_A, mAb_B, mAb_C, and pAb). We chose IgG1, the most common subclass for therapeutic agents. When acid-stressed mAb_A, mAb_B, mAb_C, and pAb were assayed using AF.2A1-AlphaScreen, all samples bound to AF.2A1 beads (Fig. 3A). Acid-stressed mAb_A showed a more than 1.5 times stronger signal than other types (mAb_B, mAb_C, and pAb). These differences in AlphaScreen signals among IgGs reflected the amount of non-native IgG generated. Though mAb_A, mAb_B, and mAb_C have an identical Fc region, not all the stressed IgG were converted to non-native IgG depending on the types of IgG. Imamura and Honda suggested that some fraction of the stressed IgG refolded to native form during neutralization^4,5^. Therefore, it is reasonable to consider that the amount of generated non-native IgG varies depending on the type of IgG, even when suffering the same stress.

Figure 3B shows the relative intensity of the reactivity between AF.2A1 and non-native IgG, normalized by setting the maximum AlphaScreen signal as 100%. The ED50 of acid-stressed mAb_A, mAb_B, mAb_C, and pAb were 136 ± 14 μg/ml, 179 ± 17 μg/ml, 125 ± 11 μg/ml, and 132 ± 7 μg/ml, respectively. The amount of stressed IgG spiked is shown in the horizontal axes, because it is difficult to estimate the actual amount of non-native IgG in a reaction mixture. Thus, ED50 did not correspond the actual ED50, because not all stressed IgG was converted to non-native structures. These apparent ED50 values were observed within a similar range, even though the actual concentration of non-native IgG was not determined. This indicates that the proposed method is applicable to practical uses. The relative comparison can be performed under the same measurement conditions even if different IgG samples are used. The results also suggest that the AF.2A1-AlphaScreen technique is capable of analyzing IgGs with different variable regions as long as the Fc region is the same. Because pAb from human serum consists of many IgG molecules with different specificities and epitope affinities, the aggregation mechanisms are not simple, and the nature of aggregates caused by stress should be much more diverse than that of mAbs. Nevertheless, the apparent ED50 of pAb and mAbs were observed within a similar range, indicating that it is possible to use pAb, which has advantages in availability and cost, as a reference material for comparative measurements with the AF.2A1-AlphaScreen technique.

### Comparing the stress responses of four types of IgG

**Figure 4:**
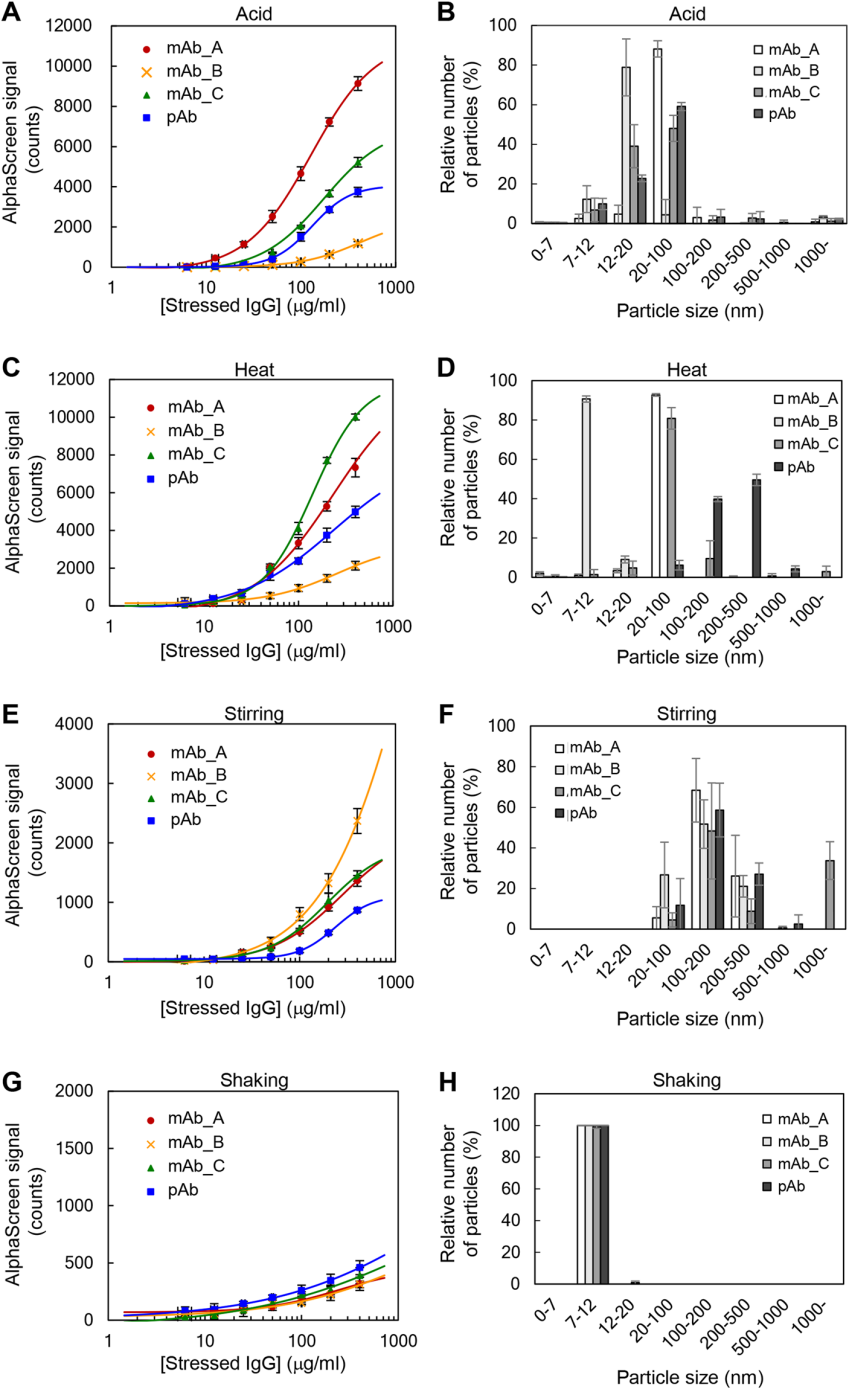
Correlation between luminescence intensity and particle diameter. (**A**) Acid-stressed IgG detected by the AF.2A1-AlphaScreen technique. (**B**) Acid-stressed IgG analyzed by DLS. (**C**) Heat-stressed IgG (70 °C, 10 min) detected by the AF.2A1-AlphaScreen technique. (**D**) Heat-stressed IgG (70 °C, 10 min) analyzed by DLS. (**E**) Stirring-stressed IgG (25 °C, 200 rpm, 5 h) detected by the AF.2A1-AlphaScreen technique. (**F**) Stirring-stressed IgG (25 °C, 200 rpm, 5 h) analyzed by DLS. (**G**) Shake-stressed IgG (4 °C, 200 rpm, 2 weeks) detected by the AF.2A1-AlphaScreen technique. (**H**) Shake-stressed IgG (4 °C, 200 rpm, 2 weeks) analyzed by DLS. Three independent experiments were performed, and the data were presented as mean ± SD.

Therapeutic IgG is subjected to various stresses in the processes of manufacturing, storage, and delivery^24^. Previous studies analyzed the mechanisms of IgG aggregation under severe conditions, such as acidic pH^25–27^, high temperature^26–29^, vigorous mechanical stress (e.g., shaking and stirring^30^), freeze/thaw^26,27^, UV light^31^, reduction^32^, oxidation^26,27^, and adsorption on solid surfaces^33^. To examine whether our technique detects non-native IgG induced by different kinds of stresses, we focused on acid (pH 2.0), heat (70 °C, 10 min), stirring (25 °C, 200 rpm, 5 h), and shaking (4 °C, 200 rpm, 2 weeks) conditions as typical examples. As shown in Fig. 4A, C, and E, the AlphaScreen signals in acid, heat, and stirring conditions gradually increased in parallel with the amount of stressed IgG. In contrast, the AlphaScreen signal was not detected for shaking stress (Fig. 4G). In addition, different AlphaScreen signals resulted from different stresses among the four types of IgG. Three samples, mAb_A, mAb_B, and mAb_C, showed the highest AlphaScreen signals under acid, stirring, and heat stresses, respectively, whereas pAb reached the highest signal under heat stress. The intensity of the AlphaScreen signal varied depending on the kind of stress and type of IgG, the reasons of which are discussed later (see Discussion).

### Determining the detectable size of IgG aggregates using DLS

**Figure 5:**
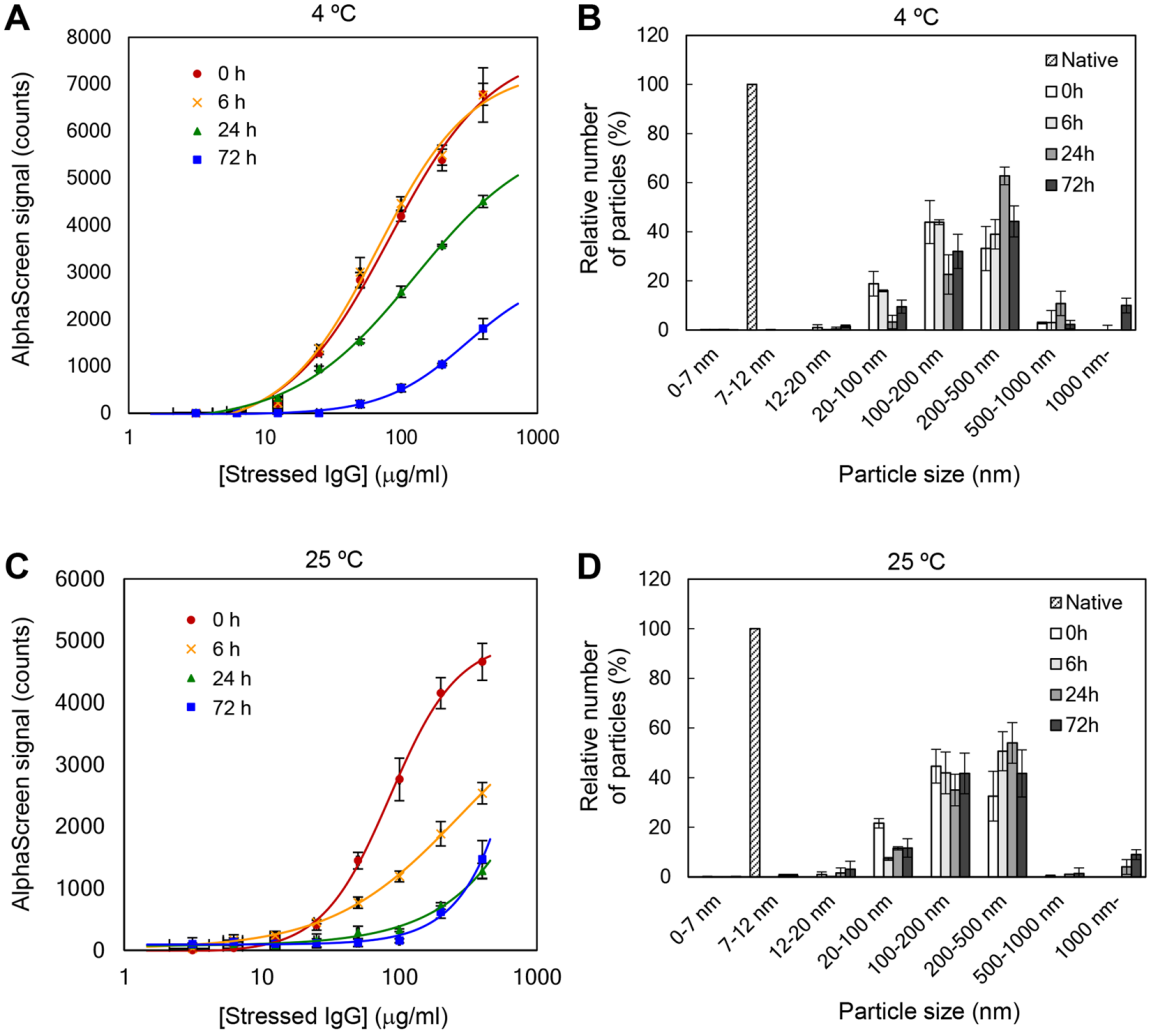
Relationship between luminescence intensity of AlphaScreen signals and particle size of non-native IgG. (**A**) Detection of acid-stressed IgG by the AF.2A1-AlphaScreen technique. The acid-stressed IgG was incubated at 4 °C for 0, 6, 24, and 72 h. Time after neutralization of the IgG solution is indicated. (**B**) Analysis of acid-stressed IgG by DLS in conditions described in (**A**). (**C**) Detection of acid-stressed IgG by the AF.2A1-AlphaScreen technique. The acid-stressed IgG was incubated at 25 °C and treated as in (**A**). (**D**) Analysis of acid-stressed IgG by DLS in conditions described in (**C**). Three independent experiments were performed, and the data were presented as mean ± SD. MAb_A was used in this experiment.

To investigate the parameters affecting the AlphaScreen signals, we focused on the size of non-native IgG aggregate, and measured particle diameter using DLS. Figure 4B, D, F, and H show the distribution of particle diameter. Particles larger than 20 nm reacted well using AF.2A1-AlphaScreen, whereas particles of shaking-stressed IgG, which did not show AlphaScreen signals (Fig. 4G), were found distributed in the range of 7–12 nm. Moreover, most mAb_B particles caused by heat stress were also distributed in the 7–12 nm range. The AlphaScreen signals of these stressed IgG molecules tended to be low as expected. In the no stress condition, native IgG monomers were distributed in the 7–12 nm range (Fig. 5B and D). These results suggest that AlphaScreen signals may differ depending on the size of aggregate particles.

To clarify whether the luminescence intensity of AlphaScreen signals changes as the particle size becomes larger, we examined the correlation between AlphaScreen signals and particle size using DLS. According to the previous study, the particle size of IgG aggregates triggered by acid-stress becomes larger in a time-dependent manner^4,5^. Based on this observation, we determined the relationship between luminescence intensity of AlphaScreen signals and time-series data on particle size. Acid-stressed IgG particles were measured 6, 24, and 72 h after neutralization. In this experiment, after neutralization, samples were incubated at 4 °C or 25 °C. When acid-stressed IgG was incubated at 4 °C, the particle size became larger in a time-dependent manner, and the AlphaScreen signal reduced remarkably after 72 h (Fig. 5A and B). In contrast, when acid-stressed IgG was incubated at 25 °C, the aggregation rate of IgG was higher than at 4 °C (Fig. 5C and D), and the AlphaScreen signal decreased 6 h after neutralization. In fact, these results are consistent with previous studies^4,5,7,34^ showing that the aggregation rate of acid-stressed IgG accelerates as the temperature increases. The AlphaScreen signal was reduced as the particle size of non-native IgG became larger (Fig. 5). The DLS results show that the AF.2A1-AlphaScreen technique is more effective for detecting aggregates smaller than ca. 500 nm (Figs 4 and 5). Therefore, this assay is useful to monitor the early stages of the IgG aggregating process.

### Determining the detectability of non-native IgG in CHO cell culture supernatant

**Figure 6:**
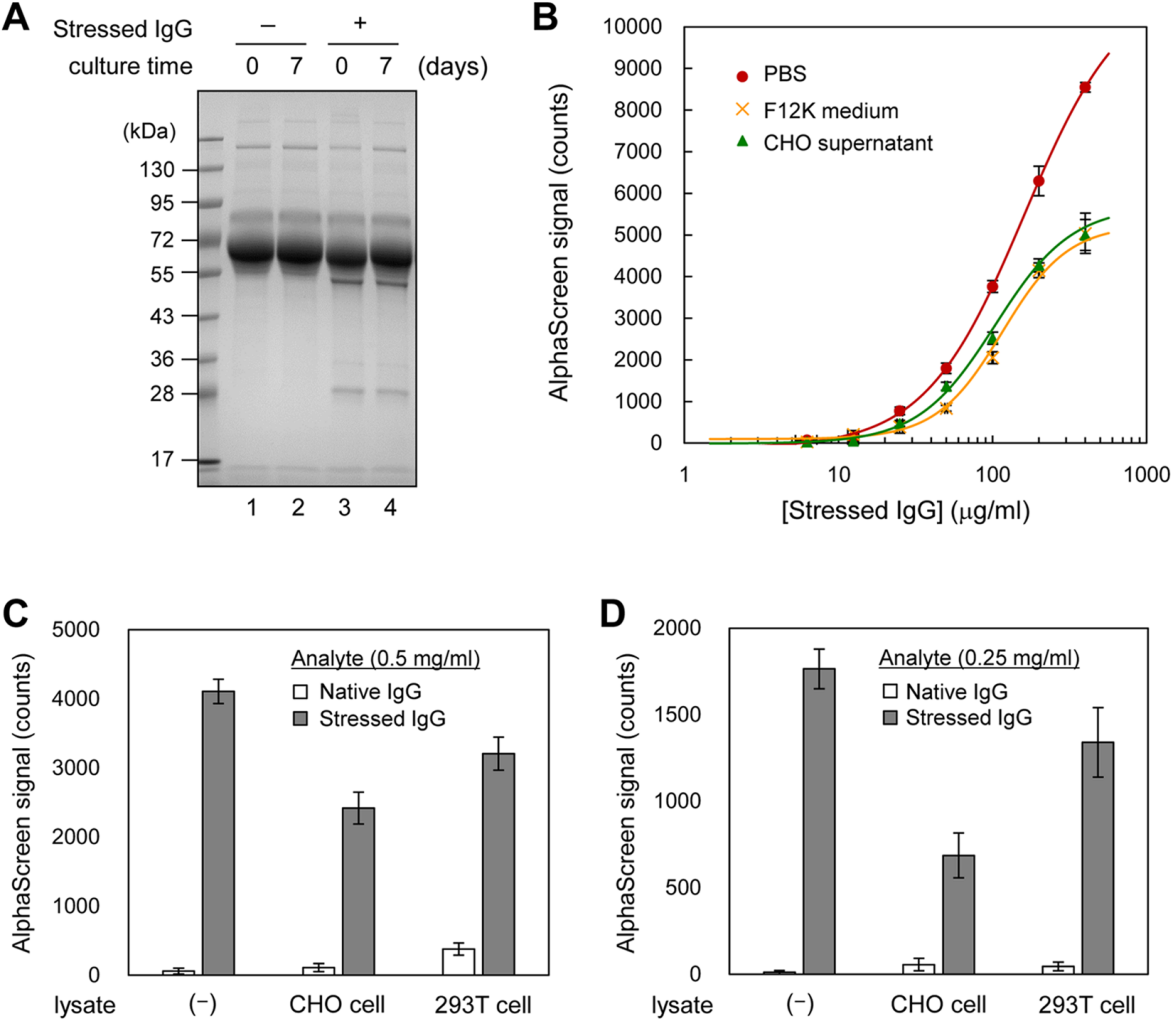
Analysis of non-native IgG in cell culture supernatant and lysate using the AF.2A1-AlphaScreen technique. (**A**) Mixture of CHO cell culture supernatant and non-native IgG. Day 0 of culture (lane 1), day 7 of culture (lane 2), day 0 of culture with 500 ng acid-stressed IgG (lane 3), and day 7 of culture with 500 ng stressed IgG (lane 4) were subjected to 12% SDS-PAGE and stained with Coomassie brilliant blue. Full-length gel image is shown in Supplementary Fig﻿ure 1. (**B**) Detection of non-native IgG in the supernatant of CHO cell culture. Acid-stressed IgG was added to PBS, F12K medium, and CHO cell culture supernatant. (**C**) Luminescence intensity of AlphaScreen signals of non-native IgG in the CHO and 293 T cell lysate. The measurement samples were prepared using acid-stressed IgG, which was added to the CHO and 293 T cell lysates. The stressed IgG concentration was 0.5 mg/ml. (**D**) Luminescence intensity of AlphaScreen signals of non-native IgG from the CHO and 293 T cell lysate, with an acid-stressed IgG concentration of 0.25 mg/ml. Otherwise conditions were as described in (**C**). Three independent experiments were performed, and the data were presented as mean ± SD. MAb_A was used in this experiment.

The manufacturing process of IgG contains several steps^35–37^, and environmental factors lead to the formation of aggregates^2,38^. Kramarczyk *et al*. reported that 20–30% of aggregates are present in IgG produced in Chinese hamster ovary (CHO) cells^39^. Therefore, techniques for analyzing non-native IgG at the cell culture stage in the manufacturing process are of particular importance. Before analyzing non-native IgG in the mammalian cell culture supernatant, we determined the amount of protein present in the supernatant. Note that CHO cells used do not produce IgG. As shown in Fig. 6, we compared the protein content between day 0 and day 7 of cell culture. F12K medium contained abundant amounts of protein, including albumin (Fig. 6A, lane 1). Coomassie brilliant blue staining showed that no significant change in the type of protein occurred in the supernatant even after day 7 of culture (Fig. 6A, lane 2). Figure 6A, lanes 3 and 4, shows the total amount of protein in the reaction mixture of AF.2A1-AlphaScreen, which contained acid-stressed IgG and medium components. A small amount of acid-stressed IgG was detected in the reaction mixture when compared with protein content in the medium (Fig. 6A).

To measure non-native IgG in the cell culture supernatant using AF.2A1-AlphaScreen, we spiked acid-stressed IgG into PBS, F12K medium, and CHO supernatant. Compared with the PBS sample, the AlphaScreen signal was reduced by 1/3 after addition of the supernatant. This is probably a result of matrix interference by colored substances in the serum with the AlphaScreen technique, in which emissions at 520–620 nm from acceptor beads are measured^20^. No significant difference was found between day 0 and day 7 (Fig. 6B). To test whether our technique detects non-native IgG in the presence of multiple types of protein, we measured the non-native IgG in CHO and human embryonic kidney 293 T (HEK293T) cell lysates. Figure 6C and D show that AF.2A1-AlphaScreen was specific to non-native IgG. Nonspecific reaction with protein in the lysate was not observed. These results indicate that AF.2A1-AlphaScreen is suitable for analyzing non-native IgG in cell culture supernatants, and therefore it is useful for monitoring it in the manufacturing process of therapeutic IgG.

## Discussion

We developed a high-throughput homogeneous assay to analyze non-native IgG in the manufacturing process of therapeutic IgG. Our results show that the technique developed has the following four advantages. First, in combination with the AlphaScreen assay, a pair of AF.2A1 proteins were used to analyze non-native IgG with high-throughput and within 90 min, as it is only necessary to add the assay components (see Methods). Moreover, because AF.2A1-AlphaScreen is available in 96-well or 384-well formats, it is easy to perform multi-analyte evaluation and automated analysis of therapeutic IgG, with the combined advantage of AlphaScreen lying in an “addition only” no-wash format. Second, AF.2A1-AlphaScreen has broad utility. AF.2A1 bound efficiently to various types of therapeutic IgG such as mAb_A, mAb_B, and mAb_C because of its ability to recognize the non-native structure of the Fc region of IgG^16^. This technique is also useful to evaluate the comparability between the prescribed original IgG and biosimilars, as the demand for biosimilars continues to increase in the pharmaceutical market^40^. Because the quality of biosimilars is significantly affected by changes in the manufacturing process and storage conditions, AF.2A1-AlphaScreen makes it possible to test a number of experimental conditions^41^. Third, AF.2A1-AlphaScreen was useful for detecting non-native IgG generated under various stresses. In the previous study, Watanabe *et al*. demonstrated that AF.2A1 does not recognize natively formed IgG, but rather non-native conformers induced by acid, heat, and reducing stresses^16,17^. We speculated that the conformational change of the AF.2A1 binding site in the Fc region caused by various stresses is common to all IgG, though various stresses might lead to a variety of morphological changes. Therefore, AF.2A1-AlphaScreen is useful at various stages of the manufacturing process where stresses are applied. Fourth, AF.2A1-AlphaScreen was able to analyze non-native IgG in the CHO cell culture supernatant. To evaluate the presence of non-native IgG in upstream process, analytical technique is required to detect non-native IgG before purification. In this study, we were able to detect non-native IgG even if other types of proteins were present in the reaction mixture. In addition, non-native IgG was detected not only in the cell culture supernatant but also in cell lysates. We believe that the AF.2A1-AlphaScreen is useful for high-throughput monitoring of the early stages of aggregation in the manufacturing process.

As mentioned in the Results section, it is important to understand that not all stressed IgG may be converted to non-native IgG. To accurately quantitate IgG aggregates, it will be necessary to develop a method to prepare pure non-native IgG stable enough to use as a reference for measurements.

Our data showed some differences in AlphaScreen signals from different types of IgG under different stress conditions (Figs 3 and 4). A possible reason is the effect of sites other than the Fc region on non-native IgG. Multiple mechanisms would lead to IgG aggregation because of the various unfolding processes occurring individually at different sites of IgG (e.g., Fv region). Because AF.2A1 recognizes non-native structures of the Fc region of IgG, aggregates driven by other sites may not be detected using AF.2A1-AlphaScreen. However, the aggregation of IgG is thought to be a stochastic reaction. Non-native IgG caused by structural changes in the Fc region would always be present at a certain predictable amount in a stressed sample. Indeed, we obtained reproducible results in detecting non-native IgG. Other possible reasons for the difference in AlphaScreen signals are the following: the number of AF.2A1 molecules immobilized on donor and acceptor beads, the number of exposed Fc regions on an aggregate particle to which AF.2A1 can bind, the number of aggregate particles in a solution, and the particle size of aggregates.

To date, most analytical methods to measure non-native IgG have been used to determine the size of aggregates. In contrast, AF.2A1 recognizes non-native IgG even in the monomeric state^17^. Considering the nature of AF.2A1, we expected it to bind to non-native IgG aggregates regardless of size. We examined the size of aggregates which could be detected by the AF.2A1-AlphaScreen technique, and our results show that it is very sensitive to small aggregates and able to detect a wide size range of aggregates with particle size of up to ca. 500 nm (Fig. 5). This size limit probably results from the distance between beads becoming too large when sandwiching a large particle of non-native IgG, which affects the detection. Another possibility is that the target structures of non-native IgG in the aggregates may change with increasing particle size. AF.2A1 may thus hardly interact with large particles. We consider the former possibility more probable at present, as it was reported in another AlphaScreen measurement^42,43^ that the distance between beads affects signal intensity. In addition, our results show that the intensities of AlphaScreen signals from acid-stressed mAb_A, mAb_B, mAb_C, and pAb varied considerably despite small differences in ED50, suggesting that the intensities are influenced by the abundance of the detectable particle sizes. In the process of manufacturing therapeutic IgG, conventional methods such as light obscuration and Coulter counter are able to analyze larger aggregates^44^. Because our technique is able to detect non-native IgG with higher sensitivity at an early stage of aggregation, it will be potentially useful in the manufacturing process for validation and quality assessment of drug products in combination with conventional methods.

As stated above, AF.2A1-AlphaScreen can be used for high-throughput measurements and may thus be employed in the various manufacturing steps of therapeutic IgG. This technique is suitable for rapid analysis of many test samples. By developing an automated analytical device with this technology, it will be useful not only for quality control of therapeutic IgG but also for screening of candidate IgGs in drug discovery research. In this study, we revealed that when non-native IgG was present in the reaction mixture, AF.2A1-AlphaScreen worked properly regardless of stress conditions. The intensities of AlphaScreen signals in the shaking stress conditions (Fig. 4G) were comparable to those of native IgG (Fig. 2A), which is consistent with the observation that the particle size under shaking stress conditions (Fig. 4H) was the same as that of native IgG (Fig. 5B). Therefore, we predicted that there was less non-native IgG in this condition. In addition, the stability of each IgG was different. This technique may also be employed in basic research for the physicochemical characterization of IgG.

## Conclusion

Our results show that the AF.2A1-AlphaScreen technique is useful for high-throughput process monitoring and quality testing in the manufacturing process of therapeutic IgG regarding the following three points. First, AF.2A1-AlphaScreen is available for the evaluation of various types of IgG because AF.2A1 recognizes the Fc region that is characteristic of IgG. Second, AF.2A1-AlphaScreen was effective in analyzing non-native IgG with particle size up to ca. 500 nm generated under different stress conditions (acid, heat, and stirring), which often happen in the manufacturing process of therapeutic IgG. Third, AF.2A1-AlphaScreen was suitable for detecting non-native IgG in cell culture supernatants. Future work includes improving detection sensitivity to reduce the possibility of immunogenicity occurring in therapeutic IgG by analyzing trace contamination of non-native IgG.

## Methods

### Materials

In a previous study, AF.2A1 was designed by modifying a 10-residue protein, chignolin, the smallest foldable polypeptide reported^45,46^. Using chignolin as a structural support, we obtained AF.2A1 by means of repetitive cycles of segment elongation and subsequent functional selection from T7 phage-displayed libraries^16^. In this study, chemically synthesized non-labeled and biotin-labeled AF.2A1 were purchased from Bio-Synthesis (Lewisville, TX, USA). IgG1 monoclonal antibodies (mAb_A, mAb_B, and mAb_C) were chosen as a model IgG. Detailed data on mAb_A, mAb_B, and mAb_C are shown in Table 1. Purified human IgG1 polyclonal antibody was purchased from Sigma-Aldrich (Darmstadt, Germany). CHO and HEK293T cells were provided by RIKEN BRC through the National Bio-Resource Project of the MEXT, Japan. CBB Stain One was purchased from Nacalai Tesque (Kyoto, Japan).

### Sample preparation

Liquid IgG and lyophilized powder of IgG were dissolved into PBS (137 mM NaCl, 2.7 mM KCl, 1.47 mM KH_2_PO_4_, and 8 mM Na_2_HPO_4_·12H_2_O) buffer at 2.4 mg/ml. Acid-stressed IgG was prepared as follows. IgG was dialyzed against 100 mM glycine-HCl (pH 2.0) with a membrane with a cut-off of 12–14 kDa (Scinova GmbH, Jena, Germany) for 16 h at 4 °C^4,5^. The dialyzed solution was stored at 4 °C before neutralization. The samples were neutralized by adding 1 volume of 1 M Tris-HCl (pH 8.0) to 5 volumes of acid-stressed IgG solution before the experiment. The term “acid stress” indicates temporal or continuous exposure of a protein to acid. Neutralization after acid exposure has been referred to in previous studies^4,5^ as “pH-shift stress”. In the present experiments, all acid-stressed IgG samples were neutralized prior to the measurements; thus, the “acid stressed IgG” referred to in this work was identical to pH-shift stressed IgG. For the other three stress conditions (heat, stirring, and shaking), IgG solutions were diluted to 2 mg/ml in PBS before stress treatment. Diluted IgG before treatment was used as negative control (native IgG). For thermal stress, IgG was incubated at 70 °C using a Dry Thermo Unit DTU-N (Taitec, Saitama, Japan) for 10 min^47^. For stirring stress, IgG was stirred in 15-cc glass vials (5 ml in each vial)^27,48^. Vials of IgG were placed in a Magnetic Stirrer HS-4DN (AS ONE, Osaka, Japan) and stirred for 5 h at 200 rpm and 25 °C. For shaking stress, IgG was agitated for 2 weeks at 200 rpm and 4 °C using a Rotary Shaker NR-20 (Taitec)^19,49^.

### Preparation of acceptor beads

AF.2A1-AlphaScreen was developed using acceptor beads (PerkinElmer, Waltham, MA, USA) conjugated to AF.2A1, biotin-AF.2A1, streptavidin-coated donor beads (PerkinElmer, Waltham, MA, USA), and stressed IgG. The conjugation of acceptor beads was performed using AF.2A1 and aldehyde-coated acceptor beads. The reaction mixture was composed of 0.1 mg of AF.2A1, 0.0625% Tween 20, 1 mg of AlphaScreen beads, and 20 mM NaBH_3_CN. The 200 μl reaction volume was completed with PBS, and the reaction mixture was incubated for 18 h at 37 °C. The reaction was stopped by the addition of 10 μl of 65 mg/ml carboxymethoxylamine solution in 0.8 M NaOH, followed by 1 h incubation at 37 °C. The carboxymethoxylamine solution was prepared before use. Beads were then washed twice by centrifugation (16,000 × *g*, 15 min, 4 °C) and resuspended in 200 μl PBS. Afterward, a third centrifugation step was performed and beads were resuspended in PBS to reach a final concentration of 5 mg/ml. The conjugated acceptor beads can be stored at 4 °C for a year.

### AlphaScreen assay

The assay was performed in a white 384-well OptiPlate (PerkinElmer, Waltham, MA, USA) containing 4 μM biotin-AF.2A1, 10 μg/ml acceptor beads conjugated to AF.2A1, and non-native IgG in AlphaScreen buffer (PBS, 1% Tween 20, and 1% bovine serum albumin). Reaction mixtures were incubated at 23 °C for 60 min. Streptavidin donor beads (20 μl at 100 μg/ml) were added (also in AlphaScreen buffer), and the plate was incubated at 23 °C for 30 min in the dark. Next, it was read on an EnSpire™ Alpha (PerkinElmer, Waltham, MA, USA) using the AlphaScreen protocol. LOD and LOQ were calculated from the calibration curve as 3.3 s/slope and 10 s/slope, respectively, where s is the standard deviation of the blank signal.

### DLS

Analysis of aggregate size distributions for native IgG and non-native IgG was performed using a DynaPro Plate Reader-II (Wyatt Technology, Santa Barbara, CA, USA) for IgG (2 mg/ml) samples. All samples and buffers were filtered through a 0.22 μm centrifugal filter (Merck, Darmstadt, Germany). Before DLS measurement, samples were equilibrated for 2 min at 25 °C and all measurements performed in triplicate. The number of runs was optimized for each sample prior to the initiation of measurements (a minimum of 10 runs was performed per measurement). The diffusion coefficient and the particle diameter by the cumulant method were calculated from the autocorrelation function using Dynamics software (Wyatt Technology)^50^.

### Cell culture and preparing of cell extract

CHO cells were cultured in Kaighn’s Modification of Ham’s F-12 Medium (American Type Culture Collection, Manassas, VA, USA) containing 10% heat-inactivated fetal calf serum. HEK293T cells were cultured in Dulbecco’s modified Eagle’s medium (Thermo Fisher Scientific, Boston, MA, USA) containing 10% heat-inactivated fetal calf serum. Cells were grown at 37 °C in a humidified incubator with a 5% CO_2_/95% air atmosphere. CHO cell culture supernatant was centrifuged at 200 × *g* for 10 min and the supernatants were stored at –80 °C. To obtain cell lysates, cells were collected before confluence and homogenized with buffer [20 mM Tris-HCl (pH 7.5), 0.05% Tween 40, 150 mM NaCl, and 1 mM phenylmethanesulfonyl fluoride]. The homogenates were centrifuged at 20,000 × *g* for 30 min, and the supernatants were harvested.

### SDS-PAGE

SDS-PAGE (12%) was performed essentially according to the instruction manual of Mini-PROTEAN TGX Precast Gels (Bio-Rad Laboratories, Hercules, CA, USA). Protein bands were visualized by staining the gels with CBB Stain One (Nacalai Tesque).

## Electronic supplementary material

Supplementary Figure 1
