# Supplementary material for: AlphaScreen-based homogeneous assay using a pair of 25-residue artificial proteins for high-throughput analysis of non-native IgG
Source: Sci Rep. 2017 Sep 29;7:12466. doi: 10.1038/s41598-017-12693-w (PMC5622108; doi:10.1038/s41598-017-12693-w)

AlphaScreen-based homogeneous assay using a pair of 25-residue artificial proteins for high-throughput analysis of non-native IgG

Yukako Senga[1], Hiroshi Imamura[1], Takamitsu Miyafusa[1], Hideki Watanabe[1], and Shinya Honda[1,*]

[1]Biomedical Research Institute, National Institute of Advanced Industrial Science and Technology (AIST), 1-1-1 Higashi, Tsukuba, Ibaraki 305-8566, Japan

*Corresponding author: s.honda@aist.go.jp

Supplementary Figure 1: Full-length gel image corresponding to cropped image in Figure 6A. We used the squared part.

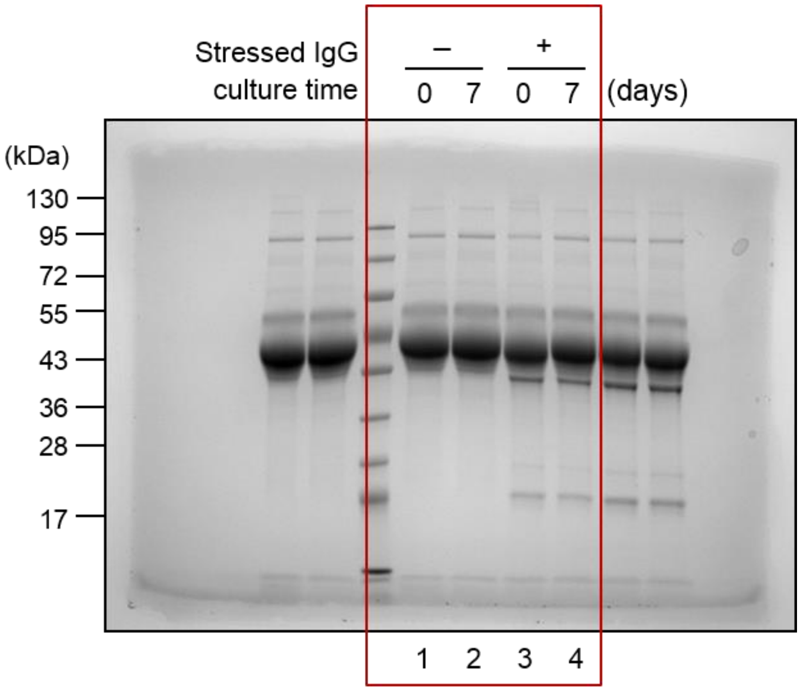

Supplement: Supplementary file 1 — Supplementary Figure 1 [file 41598_2017_12693_MOESM1_ESM.pdf]
